# Downregulation of OPA3 Is Responsible for Transforming Growth Factor-β-Induced Mitochondrial Elongation and F-Actin Rearrangement in Retinal Pigment Epithelial ARPE-19 Cells

**DOI:** 10.1371/journal.pone.0063495

**Published:** 2013-05-03

**Authors:** Seung-Wook Ryu, Jonghee Yoon, Nambin Yim, Kyungsun Choi, Chulhee Choi

**Affiliations:** 1 Department of Bio and Brain Engineering, KAIST, Daejeon, Korea; 2 KI for the Biocentury, KAIST, Daejeon, Korea; University of South Alabama, United States of America

## Abstract

Transforming growth factor-β signaling is known to be a key signaling pathway in the induction of epithelial–mesenchymal transition. However, the mechanism of TGF-β signaling in the modulation of EMT remains unclear. In this study, we found that TGF-β treatment resulted in elongation of mitochondria accompanied by induction of N-cadherin, vimentin, and F-actin in retinal pigment epithelial cells. Moreover, OPA3, which plays a crucial role in mitochondrial dynamics, was downregulated following TGF-β treatment. Suppression of TGF-β signaling using Smad2 siRNA prevented loss of OPA3 induced by TGF-β. Knockdown of OPA3 by siRNA and inducible shRNA significantly increased stress fiber levels, cell length, cell migration and mitochondrial elongation. In contrast, forced expression of OPA3 in ARPE-19 cells inhibited F-actin rearrangement and induced mitochondrial fragmentation. We also showed that Drp1 depletion increased cell length and induced rearrangement of F-actin. Depletion of Mfn1 blocked the increase in cell length during TGF-β-mediated EMT. These results collectively substantiate the involvement of mitochondrial dynamics in TGF-β-induced EMT.

## Introduction

Members of the transforming growth factor (TGF)-β family have important roles in tissue homeostasis in adults. They exert their cellular effects by forming heterotetrameric complexes of type I and type II serine/threonine kinase receptors. In the complex, the type II receptor activates the type I receptor and phosphorylates downstream effectors of the Smad family [1]–[3]. The cellular effects of TGF-β include induction of growth arrest, apoptosis, and differentiation. TGF-β overactivity has been linked to a variety of pathologic conditions including fibrosis and malignancy. Even though TGF-β was first characterized as a tumor suppressor that causes growth arrest and apoptosis, it also acts as a tumor promoter by inducing epithelial–mesenchymal transition (EMT) at later stages of tumor progression.

EMT is a cellular process whereby adherent cells disintegrate their intercellular contacts, organize their motility apparatus, and move to new locations during embryonic development and in invasive cancers and fibrotic tissues [4], [5]. TGF-β signaling is considered a very potent inducer of EMT in essentially every epithelial tissue. Activation of a Smad signaling pathway consisting of Snail1, Snail2/Slug, Smads, and HDAC6 by TGF-β is required for the establishment of EMT. Non-Smad signaling cascades involving Par3, Par6, Rho GTPase, Src, FAK kinase, JNK, and p38 MAPK have been shown to interact with canonical Smad signaling to promote EMT processes [6]–[9]. In addition to common signaling effectors of TGF-β, the modes of action of new mediators of the EMT process in response to TGF-β remain to be more firmly elucidated to provide fresh information about how TGF-β regulates cancer and fibrosis progression via EMT. In this study, we showed that mitochondrial dynamics are involved in TGF-β-induced EMT.

## Materials and Methods

### Reagents

Polyclonal antibodies specific for OPA3 [10], Mfn1 and Mfn2 were raised against the GST-fused partial protein. Antibodies against OPA1, Drp1 and Tom20 were purchased from BD Biosciences. Antibodies against Vimentin and GAPDH were from Ab Frontier. Fluor 594-conjugated goat anti-mouse and goat anti-rabbit IgGs and Fluor 488-conjugated goat anti-mouse and goat anti-rabbit IgGs were purchased from Molecular Probes. Horseradish peroxidase (HRP)-conjugated secondary antibodies were purchased from Amersham. Proteinase K and anti-actin antibody were from Sigma. Digitonin was purchased from Calbiochem.

### Expression constructs, cell culture, and transfection

OPA3 cDNA (GenBank accession no. NM_025136) was amplified by PCR. Wild-type OPA3 was amplified using specific primers, digested with EcoR1 and Sal1, and then ligated into pEYFP-N1 plasmid (Clontech) [10]. pEYFP-mito (mito-YFP) and pDsRed-mito (Clontech) were used as mitochondrial controls. The target sequence for OPA3 siRNA and OPA3 shRNA was 5′-AGCAAGCCGCTTGCCAACCGTATTA-3′ (OPA3 siRNA). Drp1, Mfn1 and Smad2 siRNAs were purchased from Bioneer (Daejeon, Korea). One day after cells were transfected with these siRNAs, the medium was changed and the cells were grown for a further 2 days.

For long-term suppression of OPA3 expression using shRNA, the region encoding the shRNA was subcloned into the *Xho*I and *Hind*III sites of pSingle-tTs-shRNA (tTs-OPA3 shRNA; Clontech). The target sequence for OPA3 was 5′-AGCAAGCCGCTTGCCAACC-3′. One day after transfection with the tTs-OPA3 shRNA construct, HeLa cells were grown in Dulbecco's complete medium containing 1 mg/mL G418 for 3 days and then in Dulbecco's complete medium containing 600 µg/mL G418 for an additional 6 days to select stable transfectants. Knockdown of the target gene is provided by a tetracycline-inducible system that responds to the presence of tetracycline or its more stable derivative, doxycycline (Dox; Clontech).

ARPE-19 cells (CRL-2302) were obtained from American Type Culture Collection. ARPE-19 cells were grown in DMEM/F12 medium (Gibco-BRL) supplemented with 100 U/mL penicillin and 100 µg/mL streptomycin (JBI, Daegu, Korea) [11]. The cells were transfected using Effectene reagent (Qiagen) and RNAiMax reagent (Invitrogen).

### Analysis of gene expression

Cell lysates were analyzed by Western blotting. SDS-PAGE was carried out using 12% polyacrylamide gels (Bio-Rad). The separated proteins were electroblotted onto nitrocellulose or polyvinylidene fluoride (PVDF) membranes (Invitrogen).

Total RNA was extracted from the cultured cells according to the manufacture's protocol (Qiagen), and 1 µg of total RNA was used for RT-PCR (Applied Biosystems, Foster, USA). Quantification of mRNA was performed using StepOne Real-Time PCR system (Applied Biosystems). Expression levels were normalized by an endogenous control, GAPDH. The following gene-specific primers were used for qPCR: human OPA3 sense 5′-CGCCGAAGCGAGTTCTTC-3′ and antisense 5′-TCTCCACCCAGTGATACAGTTGA-3′; human Mfn1 sense 5′-AGGATTGGCGTCCGTTACAT-3′ and antisense 5′-TTCCAAATCACTCCTCCAACAA-3′; human Drp1 sense 5′-TGCCAGCCAGTCCACAAA-3′ and antisense 5′-GAGCAGATAGTTTTCGTGCAACA-3′; human GAPDH sense 5′-ATGGGGAAGGTGAAGGTCG-3′ and antisense 5′-GGGGTCATTGATGGCAACAATA-3′. The data were quantified with the comparative threshold cycle (*Ct*) method for relative gene expression.

### Cell migration assay

Cell migration was evaluated by measuring the closure of a liner defect produced in a cell monolayer culture as described previously [11]. The defect was generated in a confluent culture of ARPE-19 cells by scraping with a micropipette tip. Migration distance was determined using i-Solution (iMTechnology, Korea), and the shortest distance between cells that had moved into the wounded region and their respective starting points was determined.

### Immunofluorescence

Cells grown in 2-well chamber slides were fixed by incubation with 4% paraformaldehyde for 15 min at room temperature, permeabilized by incubation with 0.15% Triton X-100 in PBS for 15 min at room temperature, and then blocked by incubation with 3% bovine serum albumin in PBS for 45 min at room temperature. The slides were incubated with the primary antibodies indicated in the figures. After washing with PBS, the slides were incubated with Alexa Fluor 488-conjugated goat anti-mouse IgG or goat anti-rabbit IgG as the secondary antibody. The slides were observed under a Zeiss LSM 510 confocal microscope using a 40× Apochromat objective (Zeiss). The excitation wavelengths for YFP, FITC, Alexa Fluor 488, and DsRed were 514, 594, 488, and 543 nm, respectively. To quantify F-actin rearrangement and cell length, cells were imaged by confocal microscopy. Fluorescence intensity and cell length were analyzed using confocal system programs. Data represent the means ± standard deviation (SD) of experiments, each with 50 cells per condition.

### Fluorescence recovery after photobleaching (FRAP)

Cells were transfected with mito-YFP. After incubation in the absence or presence of TGF-β or doxycycline, the cells were imaged using a Zeiss LSM 510. A small region of identical size (white circle, ROI) was photobleached in mito-YFP- expressing cells, using a 30.0-mV argon laser set to 488 nm with 30% laser power output and 100% transmission, until the fluorescence intensity of the region disappeared. The region was then monitored for YFP fluorescence recovery. Fluorescence intensity was normalized to the intensity of the ROI in the first image of the series, and fluorescence intensity recovery rates were plotted.

## Results

### TGF-β induces EMT and mitochondrial elongation in ARPE-19 cells

**Figure 1:**
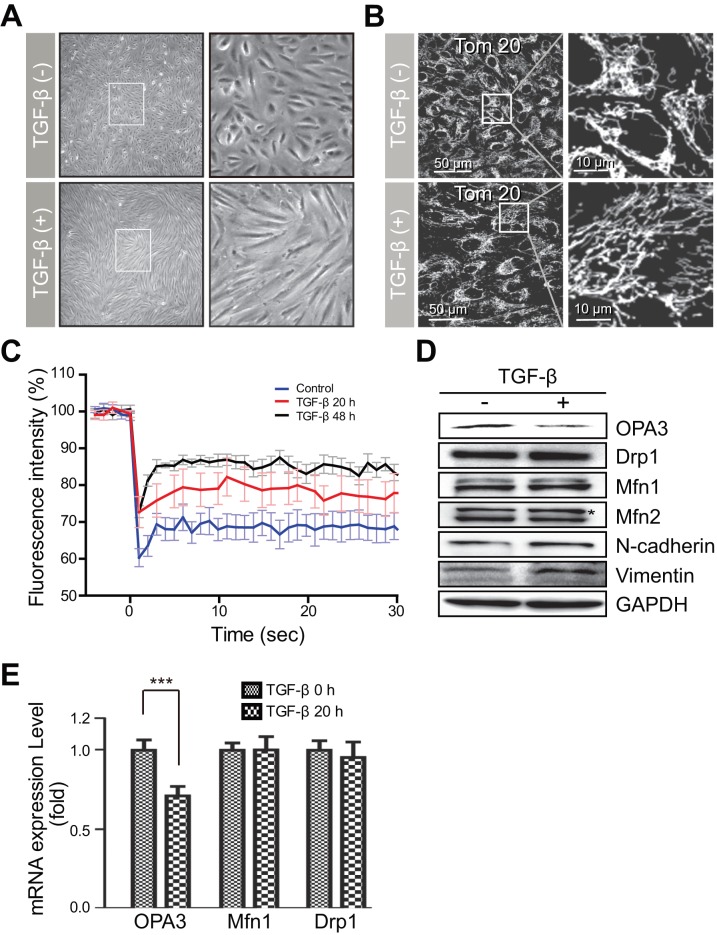
Treatment with TGF-β induced mitochondrial elongation and suppressed OPA3 expression in ARPE-19 cells. (A) Changes in cell morphology induced by TGF-β treatment. APRE-19 cells were incubated in the absence or presence of TGF-β (10 ng/mL) for 48 h. Higher magnification images of the *highlighted areas* are presented in the *panels to the right.* (B) Changes in mitochondrial morphology induced by TGF-β. After TGF-β treatment, cells were fixed and stained with anti-Tom20 antibody. Higher magnification images of the *highlighted areas* are presented in the *panels to the right*. (C) Quantification of mitochondrial fusion activity. Cells were transfected with mito-YFP. After TGF-β treatment, cells were photobleached and then monitored for recovery of mito-YFP fluorescence. Each line represents the mean of more than 30 measurements. (D and E) Reduction of OPA3 expression by TGF-β. Cells were analyzed by Western blotting (D) with the indicated antibodies and Real-time PCR (E) with the indicated quantitative primers. The Mfn2 antibody recognizes Mfn1 and Mfn2. Data are the mean ± SD of three experiments. *** *P*<0.0005.

To investigate the cellular events in TGF-β-induced EMT, ARPE-19 cells were serum-starved for 12 h and then incubated in the absence or presence of TGF-β for an additional 24 h (Fig. 1A). Along with typical EMT phenotypic changes, we observed the elongation of mitochondrial tubules following TGF-β treatment; untreated ARPE-19 cells have a concentrated mitochondrial network around the nucleus (Fig. 1B). We next measured the degree of mitochondrial elongation during TGF-β-induced EMT processes by FRAP analysis of the mitochondrial matrix-targeted yellow fluorescent protein. The fluorescence of YFP recovered more rapidly into the bleached area of the mitochondria in ARPE-19 cells after treatment with TGF-β compared to the control cells (Fig. 1C). These results clearly indicate that treatment with TGF-β induced the mitochondrial elongation along with EMT phenotypic changes.

To confirm the involvement of mitochondrial dynamics during TGF-β-induced EMT, we tested the expression of proteins related to mitochondrial morphology in ARPE-19 cells after treatment with TGF-β. Consistent with the morphologic changes, TGF-β increased the expression of the positive EMT markers α-SMA, N-cadherin and vimentin (Fig. 1D & Figure S1); the same treatment decreased the expression of the negative EMT marker protein E-cadherin (data not shown). Western blot analysis revealed that the level of optic atrophy 3 (OPA3), but not Drp1, Mfn1, or Mfn2, was decreased by TGF-β treatment in ARPE-19 cells (Fig. 1D). Consistent with this, mRNA level of OPA3, but not those of Drp1 or Mfn1, was significantly reduced by TGF-β treatment (Fig. 1E). These data suggest the involvement of proteins involved in mitochondrial dynamics, especially OPA3, in TGF-β-induced EMT.

### Smad2 signaling is essential for OPA3 regulation in TGF-β-induced EMT

**Figure 2:**
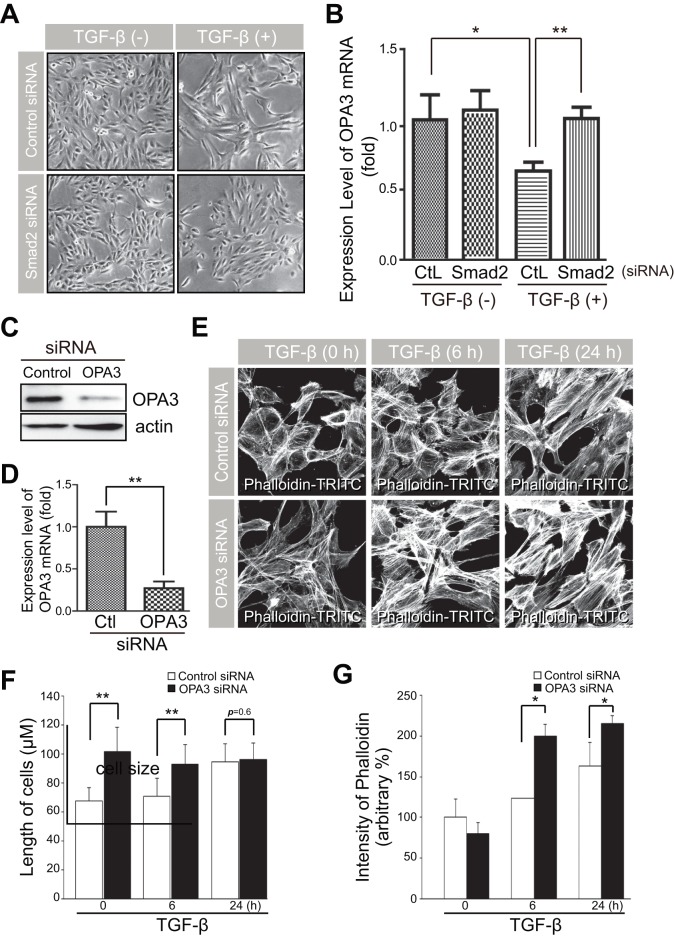
Knockdown of OPA3 induced changes in cell morphology and sensitized cells to F-actin rearrangement induced by TGF-β. (A and B) Effects of Smad2 on changes in cell morphology and OPA3 level by TGF-β treatment. (B) APRE-19 cells were transfected with Smad2 siRNA or control siRNA. Forty-eight hours after transfection, cells were incubated in the absence or presence of TGF-β for 48 h. Cells were analyzed with phase contrast microscopy (A). Real-time PCR evaluated the level of OPA3 mRNA (B). (C–G) Effects of OPA3 depletion on cell morphology and F-actin rearrangement. APRE-19 cells were transfected with OPA3 siRNA or control siRNA. Forty-eight hours after transfection, cells were treated with TGF-β for the indicated periods of time. Cells were analyzed by Western blotting (C) and Real-time PCR (D). Cells were fixed and stained with phalloidin-TRITC (E). TRITC intensity (F) and Cell lengths (G) were analyzed using confocal images. Data are the mean ± SD of three experiments, each with >50 cells per condition. *P<0.05; **P<0.005.

Smads have been well characterized as direct targets of TGF-β/TGF-β receptor signaling [2], [12]. Consistent with this, knockdown of Smad2 prevented the TGF-β-induced morphological changes (Fig. 2A). We next investigated whether blocking TGF-β signaling by Smad2 knockdown also restored the expression level of OPA3. After transient transfection of ARPE-19 cells with Smad2 siRNA, the level of Smad2 was significantly reduced compared to cells transfected with negative control siRNA (Figure S2). The basal mRNA level of OPA3 was slightly increased in cells transfected with Smad2 siRNA and dramatically reduced in control cells after TGF-β treatment compared to the control cells (Fig. 2B). Moreover, knockdown of Smad2 significantly blocked the reduction in OPA3 levels following TGF-β treatment (Fig. 2B). Consistent results were obtained for protein level of OPA3 (Figure S2). These data collectively indicate that Smad2 signaling is essential for TGF-β-induced reduction of OPA3 expression and possibly mitochondrial changes.

### Knockdown of OPA3 sensitizes cells to TGF-β-induced EMT

**Figure 3:**
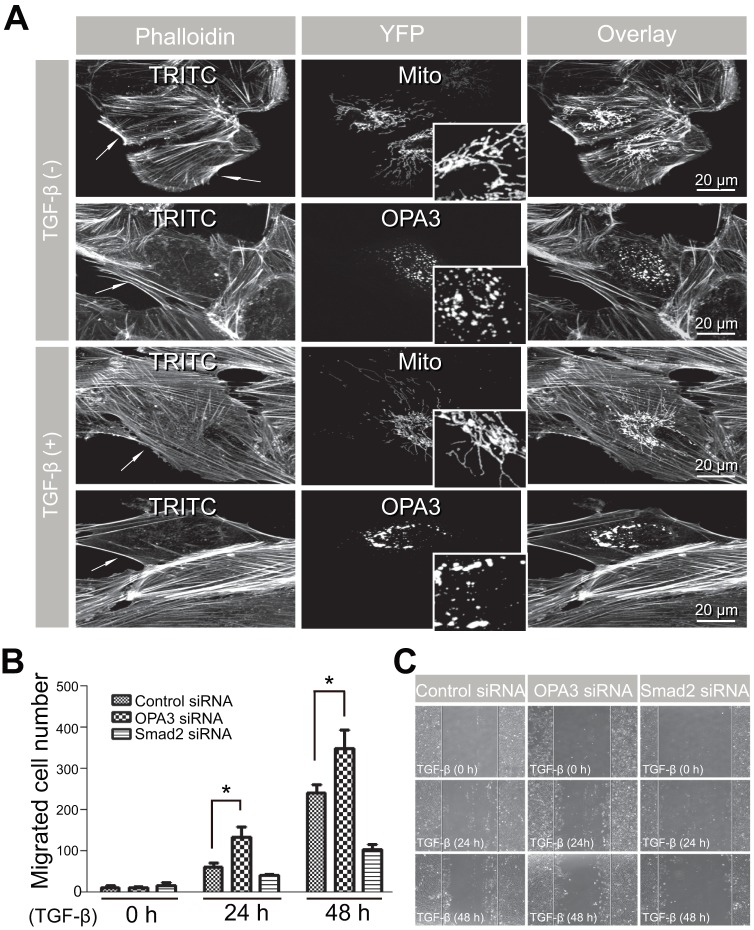
Overexpression of OPA3 prevented the rearrangement of F-actin in response to TGF-β treatment in ARPE-19 cells. (A) Inhibition of F-actin rearrangement by OPA3 overexpression. APRE-19 cells were transfected with OPA3-YFP and mito-YFP, respectively. Fifteen hours after transfection, cells were incubated in the absence or presence of TGF-β for 48 h. Cells were fixed and stained with phalloidin-TRITC. *White arrow*s indicate YFP-positive ARPE-19 cells. Higher magnification images of mitochondria are presented in the *inset panels*. (B and C) Effect of OPA3 in TGF-β-induced cell migration. Cells were transfected with the indicated siRNAs. Forty-eight hours after transfection, cells were treated with TGF-β for the indicated periods of time. The migrated cells were counted (B) and photographed (C). Data shown represent the average of three independent experiments. *P<0.05.

To elucidate the role of OPA3 in TGF-β-induced EMT, we examined the effect of OPA3 knockdown on TGF-β-induced EMT phenotypic changes. After transient transfection with OPA3 siRNA, the expression levels of OPA3 were significantly reduced compared to those in control siRNA transfectants (Fig. 2C and D). As shown in Figure 2E, typical changes in cell morphology and F-actin rearrangements (stress fibers) were observed in cells transfected with control siRNA upon treatment of TGF-β. Transient transfection with OPA3 siRNA itself induced elongation of cells similar to that observed after TGF-β treatment (Fig. 2F). Rearrangement of F-actin in response to TGF-β treatment was prominently potentiated in OPA3-knockdown cells compared to control cells (Fig. 2G). These results clearly indicate that depletion of OPA3 could sensitize cells to TGF-β signaling, leading to rearrangement of F-actin and causing cells to acquire mesenchymal cell shapes. To validate the involvement of OPA3 in rearrangement of F-actin, we examined the effect of OPA3 overexpression in ARPE-19 cells following TGF-β treatment. As shown in Figure 3A, overexpression of OPA3 induced mitochondrial fragmentation and prevented the rearrangement of F-actin in ARPE-19 cells in the absence or presence of TGF-β. These data collectively suggest that OPA3 may be involved in the rearrangement of F-actin, a major step in EMT.

We further tested the functional role of OPA3 in TGF-β-induced EMT by measuring the migratory ability of ARPE-19 cells. The treatment of TGF-β significantly increased the migration ability of control cells in Smad2-dependent manner; while the migratory activity was greatly enhanced by OPA3 knockdown (Fig. 3B and C). These results indicate that the reduced level of OPA3 is involved in TGF-β-induced cell migration of ARPE-19 cells.

### Mitochondrial elongation is required for F-actin rearrangement

**Figure 4:**
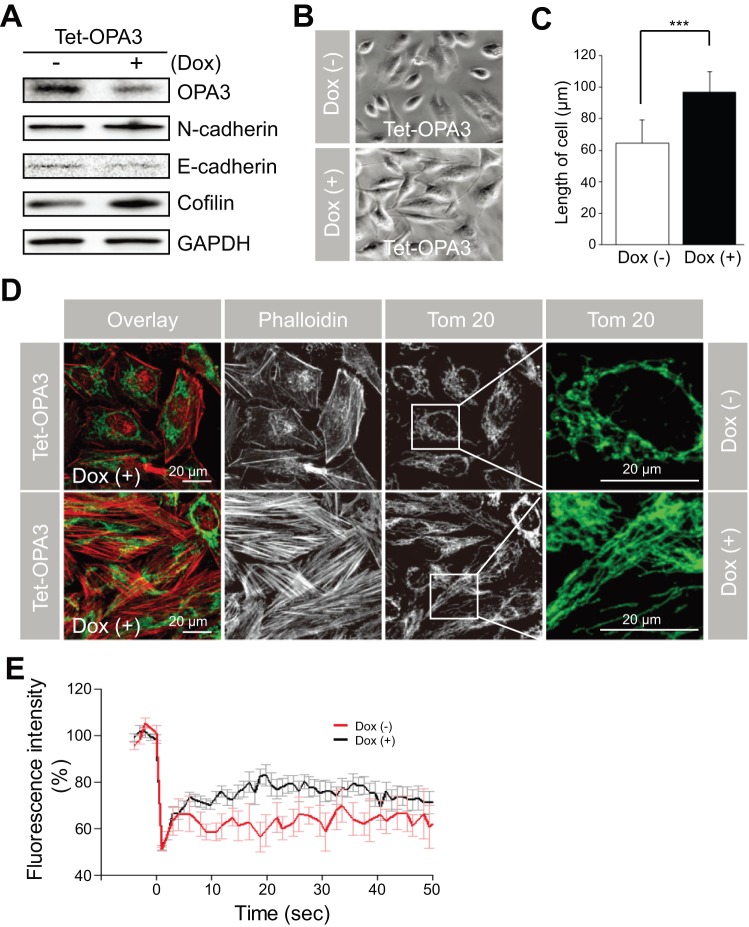
Stable knockdown of OPA3 induced the rearrangement of F-actin and mitochondrial elongation in HeLa cells. (A–D) Effect of OPA3 knockdown on changes in cell morphology. HeLa cells were transfected with an inducible OPA3 shRNA plasmid (Tet-OPA3) and then selected with G418 for 2 weeks. After selection, cells were incubated in the absence or presence of doxycycline (Dox) for 3 days. For Western blotting with the indicated antibodies (A), cells were harvested and then lysed. Cell morphology (B) and cell length (C) was analyzed using phase contrast images. Data are the mean ± SD of three experiments, each with 100 cells per condition. For confocal analysis (D), cells were fixed and stained with anti-Tom20 antibody (green) and phalloidin-TRITC (red). Higher magnification images of the *highlighted areas* are presented in the *panels to the right*. For quantification of mitochondrial fusion activity, live cells with mito-YFP were analyzed by photobleaching. Each line represents the mean of >30 measurements. ***P<0.0005.

To confirm the effect of OPA3 in other cell types, we tested HeLa cells stably expressing doxycycline (Dox)-inducible OPA3 shRNA. The level of OPA3 protein in OPA3-shRNA cells was significantly reduced by Dox treatment (Fig. 4A). Consistent with previous results in ARPE-19 cells, the mesenchymal markers such as N-cadherin and cofilin were dramatically increased; while E-cadherin was reduced in inducible OPA3-shRNA cells after Dox treatment (Fig. 4A). We also observed the EMT-like morphological changes in OPA3-shRNA cells after Dox treatment (Fig. 4B and C). For analysis of F-actin rearrangement and mitochondrial morphology, inducible OPA3-shRNA cells were stained with phalloidin and an anti-Tom20 antibody. As shown in Figure 4D, weak F-actin staining was observed at the edges of cells before Dox treatment, whereas strong F-actin staining was detected throughout the cells after Dox treatment. Consistent with our previous report [10], depletion of OPA3 induced elongation of the mitochondrial network (Fig. 4D, green) and mitochondrial fusion activity (Fig. 4E).

**Figure 5:**
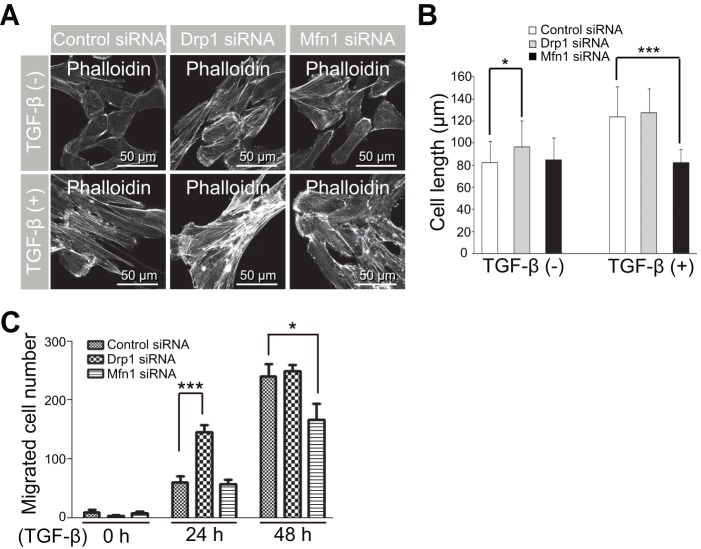
Functional changes in cells induced by knockdown of proteins involved in mitochondrial dynamics. (A and B) The mitochondrial fusion by Drp1 knockdown leads to an increase of cell length and F-actin. APRE-19 cells were transfected with Drp1 siRNA, Mfn1 siRNA, or control siRNA. Forty-eight hours after transfection, cells were treated withTGF-β for 48 h. Cells were fixed and stained with phalloidin-TRITC (A). Cell length was analyzed using confocal images (B). Data are the mean ± SD of three experiments, each with 100 cells per condition. (C) The mitochondrial fusion by Drp1 knockdown promotes cell migration induced by TGF-β. Cells were transfected with the indicated siRNAs. Forty-eight hours after transfection, cells were treated with TGF-β for the indicated periods of time. The migrated cells were counted. Data shown represent the average of three independent experiments. *P<0.05; ***P<0.0005.

To better confirm whether changes in mitochondrial morphology are required for EMT, we assessed cell length and F-actin rearrangement in ARPE-19 cells under conditions in which mitochondrial fission or fusion were selectively suppressed by transfection with Drp1 or Mfn1 siRNAs. As shown in Figure 5A and B, inhibition of mitochondrial fusion by Mfn1 siRNA significantly blocked the increase in cell length induced by TGF-β compared to control ARPE-19 cells. Like OPA3 knockdown, inhibition of mitochondrial fission by Drp1 siRNA effectively increased cell length and induced F-actin rearrangement (Fig. 5). Consistent with this, the migration ability of the cells transfected with Drp1 siRNA was significantly increased after treatment of TGF-β (Fig. 5C). Inhibition of mitochondrial fusion by Mfn1 siRNA attenuated the TGF-β-induced cell migration (Fig. 5C). These results collectively suggest that proteins involved in mitochondrial elongation may play a role in TGF-β-induced EMT.

## Discussion

In this study, we showed that mitochondrial elongation is directly involved in TGF-β-mediated EMT. A notable finding is that the downregulation of OPA3 resulted in elongated mitochondria, increased cell lengths and induced expression of EMT marker proteins. In addition, OPA3 expression was reduced during TGF-β-mediated EMT and mitochondrial shape then changed to that of an elongated tubular network. Recent studies have shown that mitochondria are dynamic structures that undergo fusion and fission events continually throughout the life of a cell. A component of the fission and fusion machinery not only affects mitochondrial biogenesis but also impedes cell cycle progression [13]–[18]. Favre *et al* (2010) showed that suppression of PNC1, which is necessary to maintain mtDNA, regulated mitochondrial biogenesis and induced EMT [19], although its exact morphological function in mitochondria remains unclear. However, mitochondrial biogenesis, the cell cycle, and reactive oxygen species have been implicated in cancer development such as EMT. Thus, it appears that defects of mitochondrial dynamics are crucially linked to cancer development.

OPA3 protein has been characterized as the mitochondrial fission machinery in cells [10]. Consistent results were obtained for the fragmentation of mitochondria when OPA3 was overexpressed and the elongation of mitochondria in response to OPA3 knockdown in ARPE-19 cells. This suggests that the function of OPA3 in mitochondrial fission is well conserved in several cell types. In the present study, we found that knockdown of OPA3 in ARPE-19 cells by OPA3 siRNA sensitized cells to TGF-β-induced EMT. Interestingly, depletion of OPA3 increased cell lengths independent of TGF-β in both cells expressing OPA3 siRNA and OPA3 shRNA. We also demonstrated that inhibition of mitochondrial fission by Drp1 knockdown increased cell length even in the absence of TGF-β in ARPE-19 cells. On the contrary, inhibition of mitochondrial fusion by Mfn1 knockdown attenuated the TGF-β –induced increase of cell length. In agreement with our study, it has been shown that inhibition of mitochondrial fission by Drp1, Fis1, and MARCH5 siRNA induced mitochondrial elongation and cell morphological changes, including enlargement, flattening, and increased cellular granularity in progression of senescence [20], [21]. Thus, cell morphological modulation induced by mitochondrial fission proteins suggests that inhibition of mitochondrial fission might serve as an inducer for EMT.

EMT is regulated by various signaling pathways at multiple stages. During progression of EMT, F-actin rearrangement mediated cellular migration. In this study, we found that knockdown of OPA3 promoted rearrangement of F-actin in a TGF-β dependent manner in APER-19 cells. We demonstrated that overexpression of OPA3 significantly inhibited rearrangement of F-actin induced by TGF-β and expression of stable OPA3 shRNA (tTs-OPA3) induced rearrangement of F-actin in HeLa cells. Furthermore, the migration ability was significantly increased in OPA3 knockdown cells compared to control cells after TGF-β treatment. Thus, our data collectively indicate that OPA3 depletion is sufficient to trigger rearrangement of F-actin. Further studies will be needed to address the signal pathway for rearrangement of F-actin by OPA3 during TGF-β-induced EMT in epithelial cells.

We demonstrated that level of OPA3 mRNA, but not Drp1 and Mfn1, is significantly reduced during TGF-β-induced EMT. We further demonstrated that inhibition of Smad2 signaling by Smad2 siRNA significantly prevented the reduction of OPA3 expression and subsequent TGF-β-induced EMT. We also showed that mitochondrial dynamics-related proteins can be controlled at the transcriptional level during TGF-β-induced EMT in Smad2-dependent manner. Although levels of proteins involved in mitochondrial dynamics (Drp1, Mfn1, and Mfn2) were not significantly altered by TGF-β-mediated EMT in this study, their knockdown induced dramatic changes in cell morphology, including cell granularity and F-actin rearrangement (Fig. 5). Non-concurrence in alternations in the expression of proteins involved in mitochondrial dynamics between morphological changes induced by TGF-β treatment and their siRNAs might be explained by protein modification. Recent studies showed that modification of proteins involved in mitochondrial dynamics (including Drp1 and Mfn1/2) is required for their functional activity [22]–[25]. Thus, further research such as studies of the phosphorylation, ubiquitination, and translocation of proteins involved in mitochondrial dynamics are necessary to clarify the role of mitochondrial dynamics in EMT.

In conclusion, we demonstrated that changes in mitochondrial dynamics, specifically induced by OPA3 are involved in TGF-β-induced EMT. The mitochondrial fusion by down-regulation of OPA3 induced an increase of mesenchymal markers and subsequent cell migration ability. Consistent with this notion, the changes in mitochondrial morphology by Drp1 and Mfn1 significantly affected cell morphology and cell migration. These findings provide new insights for the participation of mitochondrial dynamics in TGF-β-induced EMT although the detailed mechanisms involved in EMT signaling pathway still need further investigation.

## Supporting Information

Figure S1 **TGF-β induced expression of the EMT marker protein α-SMA in ARPE-19 cells.** APRE-19 cells were treated with 10 ng/mL TGF-β for the indicated periods of time. Cells were harvested, lysed, and analyzed by Western blotting with the indicated antibodies.
(EPS)

Figure S2 **Effects of Smad2 on TGF-β-mediated OPA3 expression.** APRE-19 cells were transfected with Smad2 siRNA or control siRNA. Forty-eight hours after transfection, cells were incubated in the absence or presence of 10 ng/mL TGF-β for 48 h. Cells were harvested, lysed, and analyzed by Western blotting with the indicated antibodies.
(EPS)

## References

[1] Feng XH, Derynck R (2005) Specificity and versatility in tgf-beta signaling through Smads. Annu Rev Cell Dev Biol 21: 659–693. 16212511 10.1146/annurev.cellbio.21.022404.142018

[2] Massague J, Seoane J, Wotton D (2005) Smad transcription factors. Genes Dev 19: 2783–2810. 16322555 10.1101/gad.1350705

[3] Groppe J, Hinck CS, Samavarchi-Tehrani P, Zubieta C, Schuermann JP, et al (2008) Cooperative assembly of TGF-beta superfamily signaling complexes is mediated by two disparate mechanisms and distinct modes of receptor binding. Mol Cell 29: 157–168. 18243111 10.1016/j.molcel.2007.11.039

[4] Thiery JP (2003) Epithelial-mesenchymal transitions in development and pathologies. Curr Opin Cell Biol 15: 740–746. 14644200 10.1016/j.ceb.2003.10.006

[5] Heldin CH, Landstrom M, Moustakas A (2009) Mechanism of TGF-beta signaling to growth arrest, apoptosis, and epithelial-mesenchymal transition. Curr Opin Cell Biol 21: 166–176. 19237272 10.1016/j.ceb.2009.01.021

[6] Wang X, Nie J, Zhou Q, Liu W, Zhu F, et al (2008) Downregulation of Par-3 expression and disruption of Par complex integrity by TGF-beta during the process of epithelial to mesenchymal transition in rat proximal epithelial cells. Biochim Biophys Acta 1782: 51–59. 18070611 10.1016/j.bbadis.2007.11.002

[7] Ozdamar B, Bose R, Barrios-Rodiles M, Wang HR, Zhang Y, et al (2005) Regulation of the polarity protein Par6 by TGFbeta receptors controls epithelial cell plasticity. Science 307: 1603–1609. 15761148 10.1126/science.1105718

[8] Yamashita M, Fatyol K, Jin C, Wang X, Liu Z, et al (2008) TRAF6 mediates Smad-independent activation of JNK and p38 by TGF-beta. Mol Cell 31: 918–924. 18922473 10.1016/j.molcel.2008.09.002 PMC2621323

[9] Cicchini C, Laudadio I, Citarella F, Corazzari M, Steindler C, et al (2008) TGFbeta-induced EMT requires focal adhesion kinase (FAK) signaling. Exp Cell Res 314: 143–152. 17949712 10.1016/j.yexcr.2007.09.005

[10] Ryu SW, Jeong HJ, Choi M, Karbowski M, Choi C (2010) Optic atrophy 3 as a protein of the mitochondrial outer membrane induces mitochondrial fragmentation. Cell Mol Life Sci 67: 2839–2850. 20372962 10.1007/s00018-010-0365-z PMC11115811

[11] Choi K, Lee K, Ryu SW, Im M, Kook KH, et al (2012) Pirfenidone inhibits transforming growth factor-beta1-induced fibrogenesis by blocking nuclear translocation of Smads in human retinal pigment epithelial cell line ARPE-19. Mol Vis 18: 1010–1020. 22550395 PMC3339036

[12] Massague J, Wotton D (2000) Transcriptional control by the TGF-beta/Smad signaling system. Embo J 19: 1745–1754. 10775259 10.1093/emboj/19.8.1745 PMC302010

[13] Margineantu DH, Gregory Cox W, Sundell L, Sherwood SW, Beechem JM, et al (2002) Cell cycle dependent morphology changes and associated mitochondrial DNA redistribution in mitochondria of human cell lines. Mitochondrion 1: 425–435. 16120295 10.1016/s1567-7249(02)00006-5

[14] Chen H, Chan DC (2005) Emerging functions of mammalian mitochondrial fusion and fission. Hum Mol Genet 14 Spec No. 2: R283–289. 10.1093/hmg/ddi270 16244327

[15] Arakaki N, Nishihama T, Owaki H, Kuramoto Y, Suenaga M, et al (2006) Dynamics of mitochondria during the cell cycle. Biol Pharm Bull 29: 1962–1965. 16946518 10.1248/bpb.29.1962

[16] Alirol E, Martinou JC (2006) Mitochondria and cancer: is there a morphological connection? Oncogene 25: 4706–4716. 16892084 10.1038/sj.onc.1209600

[17] Martinez-Diez M, Santamaria G, Ortega AD, Cuezva JM (2006) Biogenesis and dynamics of mitochondria during the cell cycle: significance of 3′UTRs. PLoS One 1: e107. 17205111 10.1371/journal.pone.0000107 PMC1762426

[18] Jezek P, Plecita-Hlavata L (2009) Mitochondrial reticulum network dynamics in relation to oxidative stress, redox regulation, and hypoxia. Int J Biochem Cell Biol 41: 1790–1804. 19703650 10.1016/j.biocel.2009.02.014

[19] Favre C, Zhdanov A, Leahy M, Papkovsky D, O'Connor R (2010) Mitochondrial pyrimidine nucleotide carrier (PNC1) regulates mitochondrial biogenesis and the invasive phenotype of cancer cells. Oncogene 29: 3964–3976. 20453889 10.1038/onc.2010.146

[20] Park YY, Lee S, Karbowski M, Neutzner A, Youle RJ, et al (2010) Loss of MARCH5 mitochondrial E3 ubiquitin ligase induces cellular senescence through dynamin-related protein 1 and mitofusin 1. J Cell Sci 123: 619–626. 20103533 10.1242/jcs.061481 PMC2818198

[21] Lee S, Jeong SY, Lim WC, Kim S, Park YY, et al (2007) Mitochondrial fission and fusion mediators, hFis1 and OPA1, modulate cellular senescence. J Biol Chem 282: 22977–22983. 17545159 10.1074/jbc.M700679200

[22] Gegg ME, Cooper JM, Chau KY, Rojo M, Schapira AH, et al (2010) Mitofusin 1 and mitofusin 2 are ubiquitinated in a PINK1/parkin-dependent manner upon induction of mitophagy. Hum Mol Genet 19: 4861–4870. 20871098 10.1093/hmg/ddq419 PMC3583518

[23] Chang CR, Blackstone C (2007) Drp1 phosphorylation and mitochondrial regulation. EMBO Rep 8: 1088–1089; author reply 1089–1090. 10.1038/sj.embor.7401118 PMC2267246 18059302

[24] Braschi E, Zunino R, McBride HM (2009) MAPL is a new mitochondrial SUMO E3 ligase that regulates mitochondrial fission. EMBO Rep 10: 748–754. 19407830 10.1038/embor.2009.86 PMC2727426

[25] Kar R, Mishra N, Singha PK, Venkatachalam MA, Saikumar P (2010) Mitochondrial remodeling following fission inhibition by 15d-PGJ2 involves molecular changes in mitochondrial fusion protein OPA1. Biochem Biophys Res Commun 399: 548–554. 20678484 10.1016/j.bbrc.2010.07.108 PMC2942079

